# Peritoneal Sarcoidosis

**DOI:** 10.1111/ans.70475

**Published:** 2026-01-02

**Authors:** Luiz Eduardo Correia Miranda, André Bezerra de Sena, Raphael Brito Vieira

**Affiliations:** ^1^ Hospital Oswaldo Cruz, Universidade de Pernambuco Recife Pernambuco Brazil

1

Patients presenting with weight loss and ascites of non‐portal etiology are frequently diagnosed with peritoneal carcinomatosis, a diagnosis that may be suggested by imaging modalities [1]. A 53‐year‐old woman presented to the surgical clinic with complaints of abdominal discomfort and distension over the past 6 months. She reported a 7 kg weight loss during this period. Physical examination revealed no stigmata of chronic liver disease, although moderate‐volume ascites was present. The patient denied fever, cardiorespiratory, or genitourinary complaints. Laboratory investigations were unremarkable, including normal amylase, bilirubin, and canalicular enzymes. Serum albumin was 3.8 g/dL, and ascitic fluid albumin was 3.1 g/dL, yielding a serum‐ascites albumin gradient of 0.7 g/dL. Serum angiotensin‐converting enzyme was not measured.

Ascitic fluid analysis revealed 1005 red blood cells/μL and 2034 leukocytes/μL, with a pH of 8.0. Microbiological studies, including bacterial and fungal cultures, were negative. GeneXpert polymerase chain reaction testing for tuberculosis was also negative. T2‐weighted magnetic resonance imaging of the abdomen and pelvis demonstrated massive ascites, peritoneal thickening (Figure 1A, arrow), and heterogeneous amorphous lesions scattered throughout the parietal fat and pelvis (Figure 1B, arrow), findings suggestive of peritoneal carcinomatosis. The imaging studies failed to identify a primary malignancy. Upper gastrointestinal endoscopy and colonoscopy yielded normal results.

**FIGURE 1:**
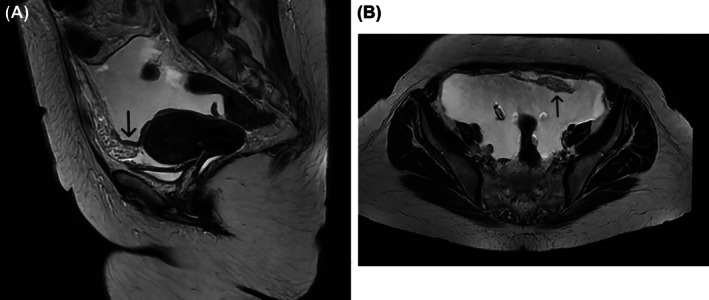
(A) T2‐weighted magnetic resonance imaging of the abdomen and pelvis showing peritoneal thickening; (B) T2‐weighted magnetic resonance imaging of the abdomen and pelvis showing heterogeneous amorphous lesions scattered throughout the parietal fat and pelvis (arrow).

Diagnostic laparoscopy revealed moderate‐volume dark citrine‐yellow ascites, with isolated or coalescent whitish plaques and isolated carmine‐red nodules extensively involving the visceral and parietal peritoneal surfaces, abdominal ligaments, and abdominal fat. The liver surface (Figure 2A) and pelvis (Figure 2B) were particularly involved by these lesions. This is a rare laparoscopic image of an uncommon disease. Histopathological examination of the peritoneal biopsies demonstrated chronic granulomatous inflammation without central necrosis (sarcoid‐type granulomas). Special staining for fungi and acid‐fast bacilli was negative, and no evidence of malignancy was identified in the specimens.

**FIGURE 2:**
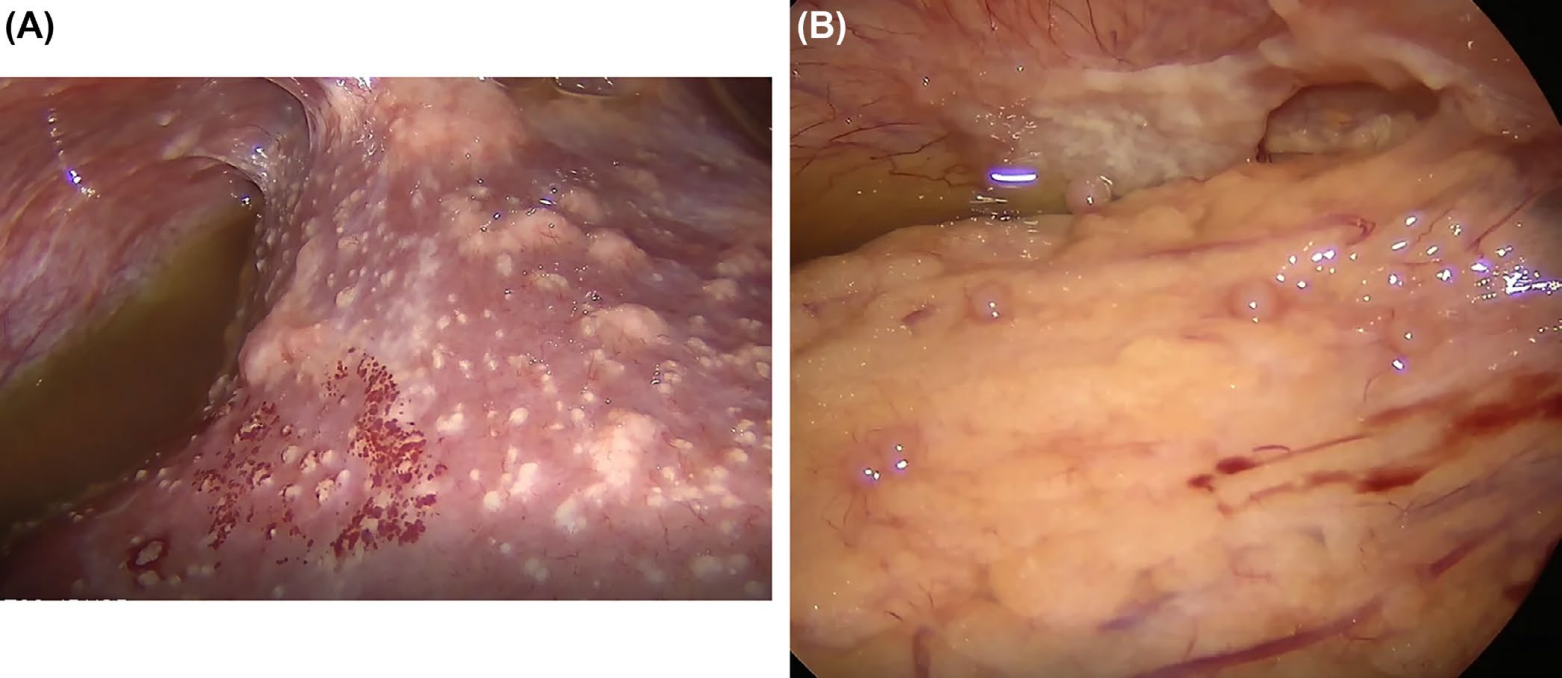
(A) Laparoscopy image showing the liver surface; (B) laparoscopy image showing the pelvis.

Sarcoidosis is a rare multisystem granulomatous disease of unknown etiology, characterized by noncaseating granulomas [2] The lungs and lymph nodes are most commonly affected. Peritoneal involvement represents an exceedingly rare manifestation, more frequently observed in women between the second and fourth decades of life [3, 4] Given that the clinical presentation and radiological findings can easily mimic peritoneal carcinomatosis or tuberculous peritonitis, histopathological confirmation through biopsy is mandatory for accurate diagnosis [1, 5].

The patient was initiated on oral corticosteroid therapy with prednisone, starting at 40 mg daily and tapering to 20 mg over 2 months. She demonstrated excellent clinical response, with complete resolution of symptoms and ascites, as well as full recovery of her baseline weight.

GeneXpert polymerase chain reaction testing for tuberculosis was also negative. T2‐weighted magnetic resonance imaging of the abdomen and pelvis demonstrated massive ascites, peritoneal thickening (Figure 1A), and heterogeneous amorphous lesions scattered throughout the parietal fat and pelvis (Figure 1B), findings suggestive of peritoneal carcinomatosis.

## Author Contributions

L.E.C.M.: conceptualization, surgical procedure, manuscript writing and revision. A.B.S.: image collection, literature review. R.B.V.: data collection, image acquisition, manuscript revision. All authors approved the final version of the manuscript.

## Funding

The authors have nothing to report.

## Ethics Statement

Written informed consent was obtained from the patient for publication of this case report and accompanying images. Patient confidentiality has been maintained, and no identifying information is disclosed in this manuscript.

## Conflicts of Interest

The authors declare no conflicts of interest.

## Data Availability

The data that support the findings of this study are available from the corresponding author upon reasonable request.
